# Robot-assisted percutaneous screw fixation in the treatment of navicular fracture

**DOI:** 10.3389/fsurg.2022.1049455

**Published:** 2023-01-05

**Authors:** Cheng Wang, Shaoling Fu, Xueqian Li, Jiazheng Wang, Chenglin Wu, Jieyuan Zhang, Guoxun Song, Wenqi Gu, Zhongmin Shi

**Affiliations:** Department of Orthopaedic Surgery, Shanghai Sixth People's Hospital, Shanghai, China

**Keywords:** navicular, fracture, percutaneous screw placement, minimally invasive, robot-assisted, robot surgery

## Abstract

**Background:**

Long recovery time, large scar, postoperative swelling and pain are possible side effects of open reduction internal fixation (ORIF) for tarsal navicular fractures. Early exercise instruction is made possible by the use of an intraoperative robot-assisted percutaneous invasive closed reduction internal fixation. The goal of the trial was to determine whether percutaneous screw internal fixation with robot assistance might be used to treat navicular fractures.

**Methods:**

27 patients with navicular fractures had surgical treatment between June 2019 and December 2021. Of those, 20 instances were treated with ORIF, while 7 cases had robot-assisted percutaneous screw internal fixation. At the final follow-up, the American Orthopaedic Foot & Ankle Society (AOFAS) hindfoot score and the visual analogue scale (VAS) score were compared to determine outcomes and function.

**Results:**

Follow-up was obtained in all 27 patients after surgery, with a mean follow-up time of 21.81 months, ranging from 15 to 29 months . In the 7 instances of robot-assisted group, percutaneous guide wire insertion and screw placement only needed one attempt and the depth and position of the implant were both satisfactory. In the ORIF group, there were two patients who sustained cutaneous nerve injuries. The AOFAS score and the VAS score of the group receiving robot-assisted navigation percutaneous screw fixation were 92.25 ± 2.22 and 0.75 ± 0.25 respectively at the last follow-up, while 82.25 ± 7.15 and 0.50 ± 0.29 were the respective values for the ORIF group.

**Conclusion:**

Intraoperative robot-assisted percutaneous closed reduction internal fixation for tarsal navicular fractures can accomplish exact localization of fracture site, reduce soft tissue damage and operative time. According to current view, this method offers fewer complications, a faster recovery after surgery, and more patient satisfaction.

## Introduction

Tarsal navicular fractures are uncommon, with fractures in this region accounting for approximately 0.45% of systemic fractures, 5.1% of all foot fractures, and 35% of all midfoot fractures (1, 2). The navicular bone carries most of the axial load during hindfoot movement and is therefore an important component of not only the Chopart joint but also a key structure in the medial column of the midfoot (3, 4). The navicular bone is often susceptible to shear forces resulting in fractures due to insufficient blood supply in the middle third of the navicular body and violent conduction of the attached soft tissues such as the talofibular ligament, part of the deltoid ligament, and the posterior tibial tendon (5). Inadequate blood supply also increases the risk of non-union, delayed healing, and ischemic osteonecrosis (5, 6). Sangeorzan classified navicular fractures into four main types based on the direction of the fracture line, the direction of foot displacement, and the involvement of the surrounding joints: navicular tuberosity fracture, navicular avulsion fracture, navicular stress fracture, and navicular body fracture, and further subdivided the navicular body fracture into three types (7). Avulsion fractures of the navicular bone account for about 50% of the four types of navicular fractures, and other types of high-energy injuries such as traffic accidents and falls are becoming more frequent (3, 4, 8–10). Schmid classified the fractures of the navicular bone into types 1 to 3 according to the degree of involvement of the talocalcaneal joint, referring to fractures of both parts of the navicular body, comminuted fractures and dislocation of the periprosthetic joint/talar head fractures, respectively (11). CT examinations can better demonstrate the type of fracture, the extent of articular surface destruction and the remaining combined injuries, while excluding pars distalis and anatomic variants (4, 12).

Displaced navicular fractures, navicular body fractures, and combined talocrural and navicular-cuneiform joint destruction require consideration of surgical treatment (4). The treatment of navicular fractures include Open reduction internal fixation (ORIF) and closed reduction internal fixation. ORIF is currently the mainstream surgical treatment for navicular fractures, but the large incision with high tension of surrounding soft tissues and ligaments and joint capsule requires further debridement, and therefore there are problems such as incision-related complications, impaired blood flow, and poor healing (13). Closed reduction is based on the mechanism of reverse injury and can achieve satisfactory anatomical reduction. The position, angle and length of the screw during closed reduction internal fixation affect the final result of surgical reduction and fixation of the navicular fracture. Due to the complex anatomy of the navicular bone and the narrow space for internal fixation placement, achieving accurate percutaneous screw internal fixation of navicular fractures is not an easy task. The current problems lie in the lack of repositioning precision, high experience requirements, and long learning curve (3, 4, 10).

The purpose of this study is to report the outcomes of 27 patients with tarsal navicular fractures who came to our institution for surgical treatment with robot-assisted closed reduction percutaneous screw internal fixation and ORIF, respectively. We compared these two treatment modalities analyzed and described the technical aspects and feasibility of robot-assisted closed reduction percutaneous screw fixation to provide guidance for the surgical treatment modality of tarsal navicular fractures.

## Methods

### Patients and study design

Inclusion criteria: (1) x-ray and CT confirmed the diagnosis of navicular fracture; (2) closed fracture; (3) good skin condition of the affected foot. Exclusion criteria: (1) comminuted fracture of the navicular bone, resulting in cases where talocalcaneal fusion must be considered; (2) open fracture; (3) pathological fracture; (4) combined diabetes mellitus, acute infection; (5) multiple midfoot injuries; (6) incomplete follow-up data, follow-up time <6 months.

**Figure 1:**
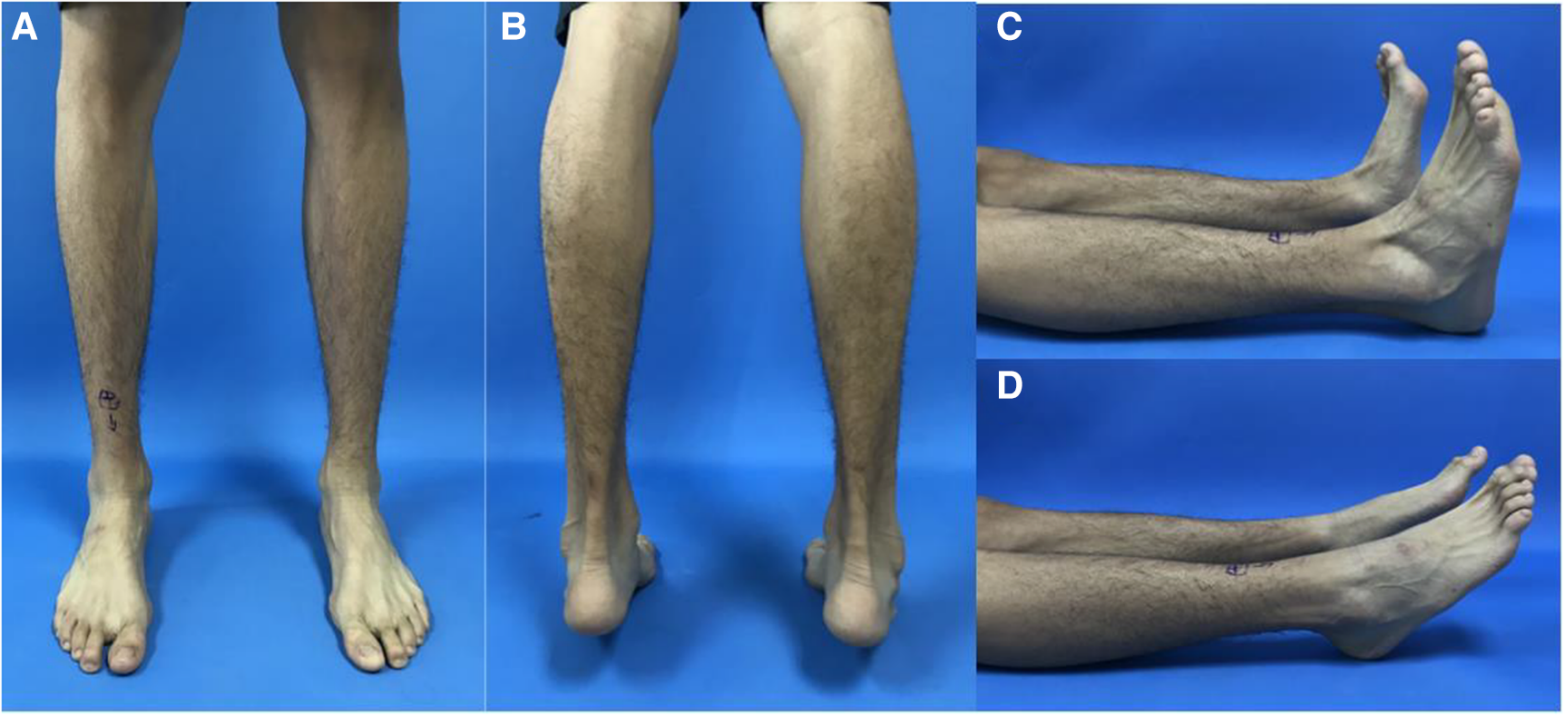
Preoperative imaging showed that the bearing capacity of the medial column of the affected foot was weakened, the posterior foot was slightly varus, the posterior foot force line was slightly varus biased, the dorsiflexion activity was slightly limited, and the plantarflexion activity was not limited. (**A**) Front view standing on two feet; (**B**) rear view with tiptoe feet; (**C**) lateral view of dorsal extension of both feet; (**D**) lateral view of plantar flexion of both feet.

A total of 27 patients with navicular fractures were included in the study period, including 12 males and 15 females; age ranged from 14 to 48 years, with an average of 35.07 years; 12 cases were left foot, 14 cases were right foot, and 1 case was bipedal; the causes of injury included 10 cases of sprain, 13 cases of fall injury, and 4 cases of traffic injury. The patients’ symptoms were pain on the dorsal side of the midfoot, and the pain increased when standing and bearing weight. The dorsum of the midfoot was painful (+) and slightly swollen. The active dorsiflexion and plantar flexion of the affected limb were slightly limited, and the toe movement was possible (Figure 1). The fractures of the navicular bone were classified according to the Sangeorzan classification as type 2 navicular tuberosity fractures in 4 cases and type 3 navicular body fractures in 23 cases according to preoperative imaging data. The study was approved by the Medical Ethics Committee of the Sixth People's Hospital of Shanghai Jiao Tong University, and complied with the code of ethics of the World Medical Association (Declaration of Helsinki). Written consent was obtained from all participants.

### Preoperative planning

**Figure 2:**
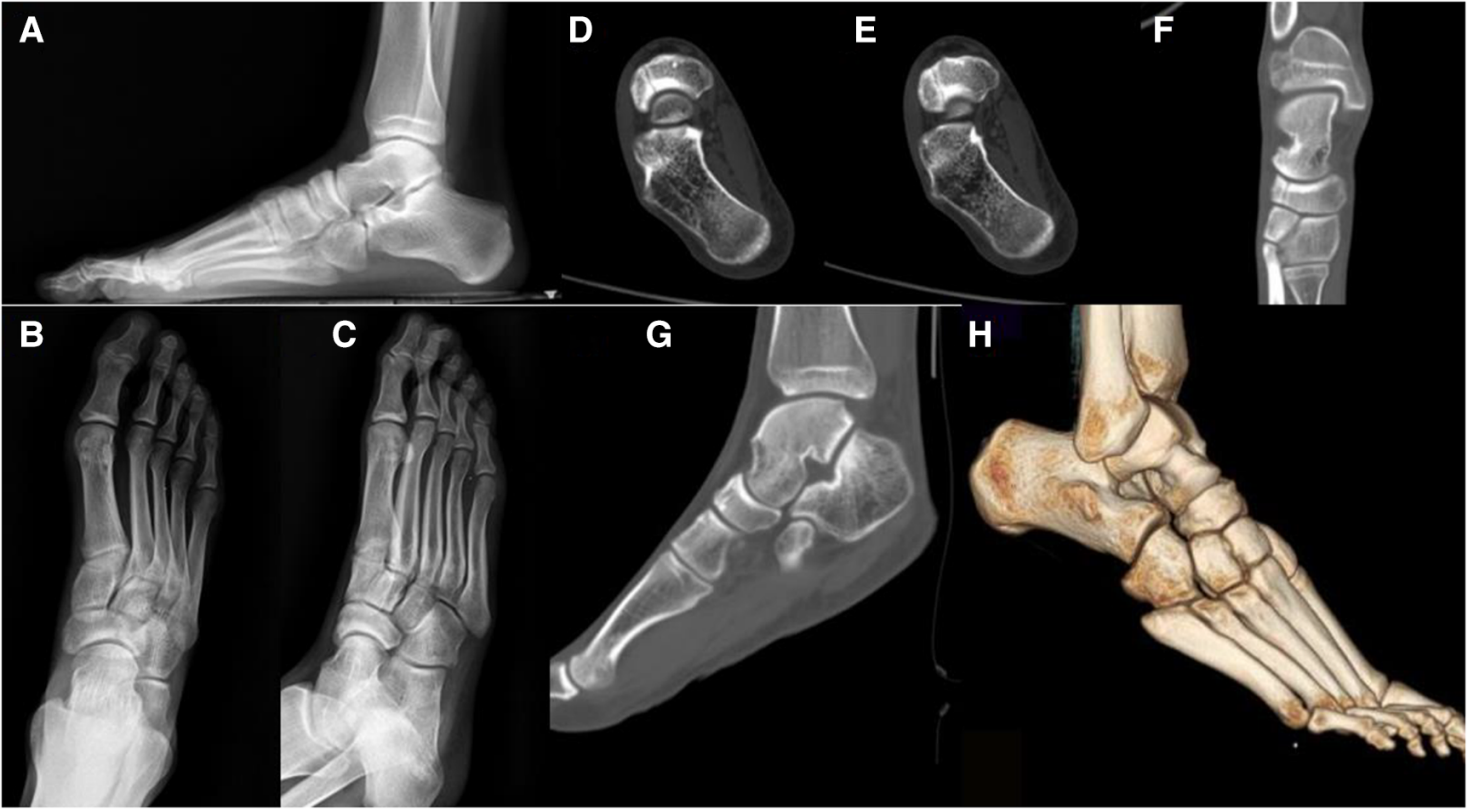
Preoperative imaging data of the right foot showed a tarsal navicular fracture of the right foot (Sangeorzan Type III.2). The fracture line extended from dorsolateral to plantar, and the medial column was stable. (**A**) Weight-bearing lateral radiographs of the right foot; (**B**) anteroposterior x-ray of the right foot; (**C**) oblique radiography of the right foot; (**D,E**) CT axial view of the right foot; (**F**) CT coronal view of the right foot; (**G**) CT sagittal view of the right foot; (**H**) 3D CT reconstruction of the right foot.

All patients had preoperative frontal, oblique and lateral x-rays, Weight-bearing x-rays and 3D CT examinations. Then we used the picture archiving and communication system to obtain 3D reconstructed images of the navicular bone to initially simulate the repositioning situation, and the position, length and direction of the screws (Figure 2). Imaging data were independently evaluated by 2 experienced orthopeadic surgeons.

### Operative technique

• The use of intraoperative robot-assisted closed-reposition percutaneous screw internal fixation for navicular fractures of the foot was performed as follows.

Advance preparation of the robot-assisted surgical system. The robot-assisted surgical system consists of TINAVI intelligent orthopaedic surgical robot (TINAVI Medical Technologies, Beijing, China), which includes robotic arm mainframe, optical tracking system, main control dolly, as well as an immediate intraoperative 3D imaging system- Artis zeego (ARCADIS Orbic 3D; Siemens, Erlangen, Germany), and other related accessories, such as navigation indicators.

**Figure 3:**
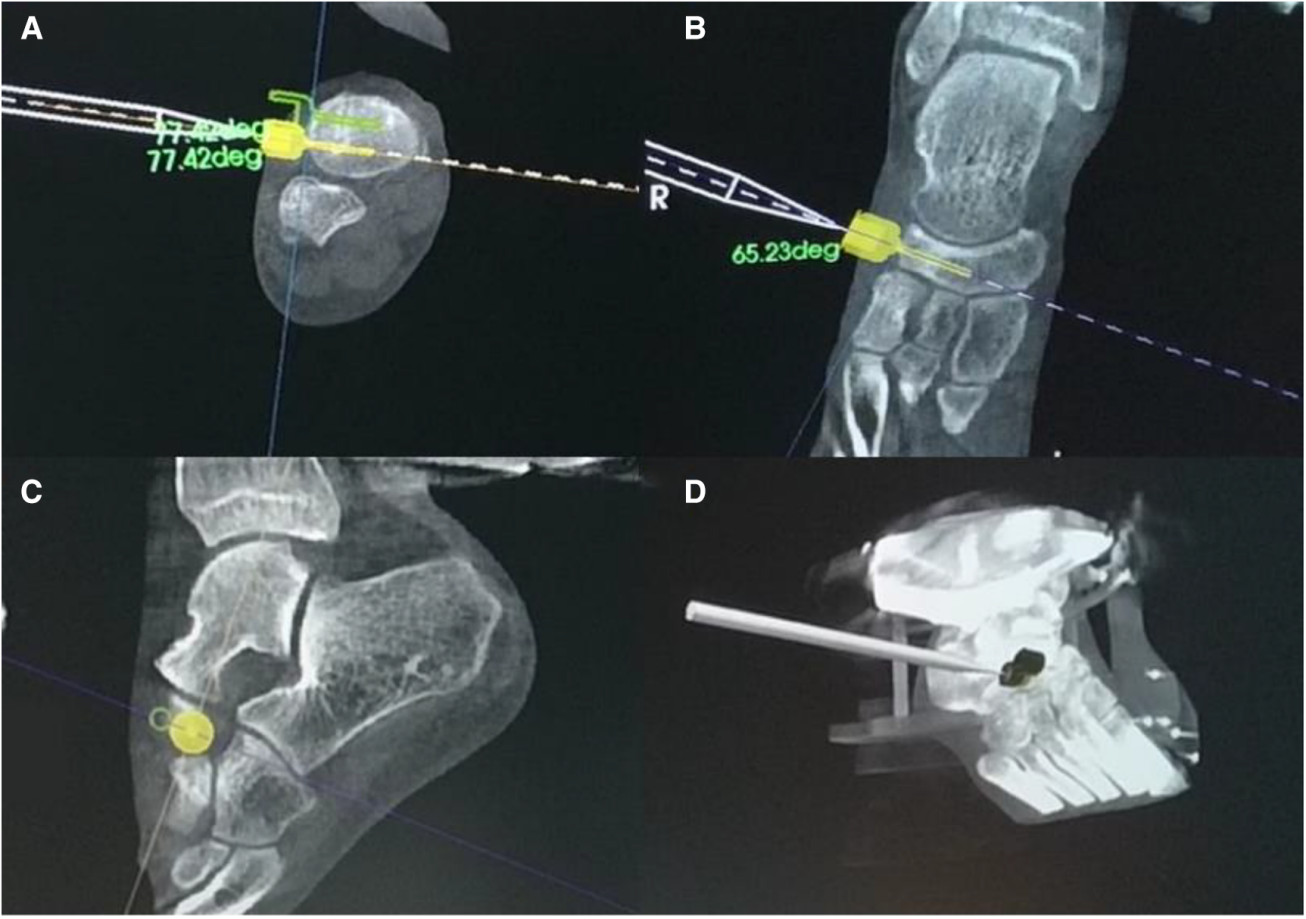
TINAVI intelligent orthopedic robot navigated during the operation and calculated the screw placement trajectory according to three-dimensional CT. (**A**) CT axial view of the right foot; (**B**) CT coronal view of the right foot; (**C**) CT sagittal view of the right foot; (**D**) 3D CT reconstruction of the right foot.

**Figure 4:**
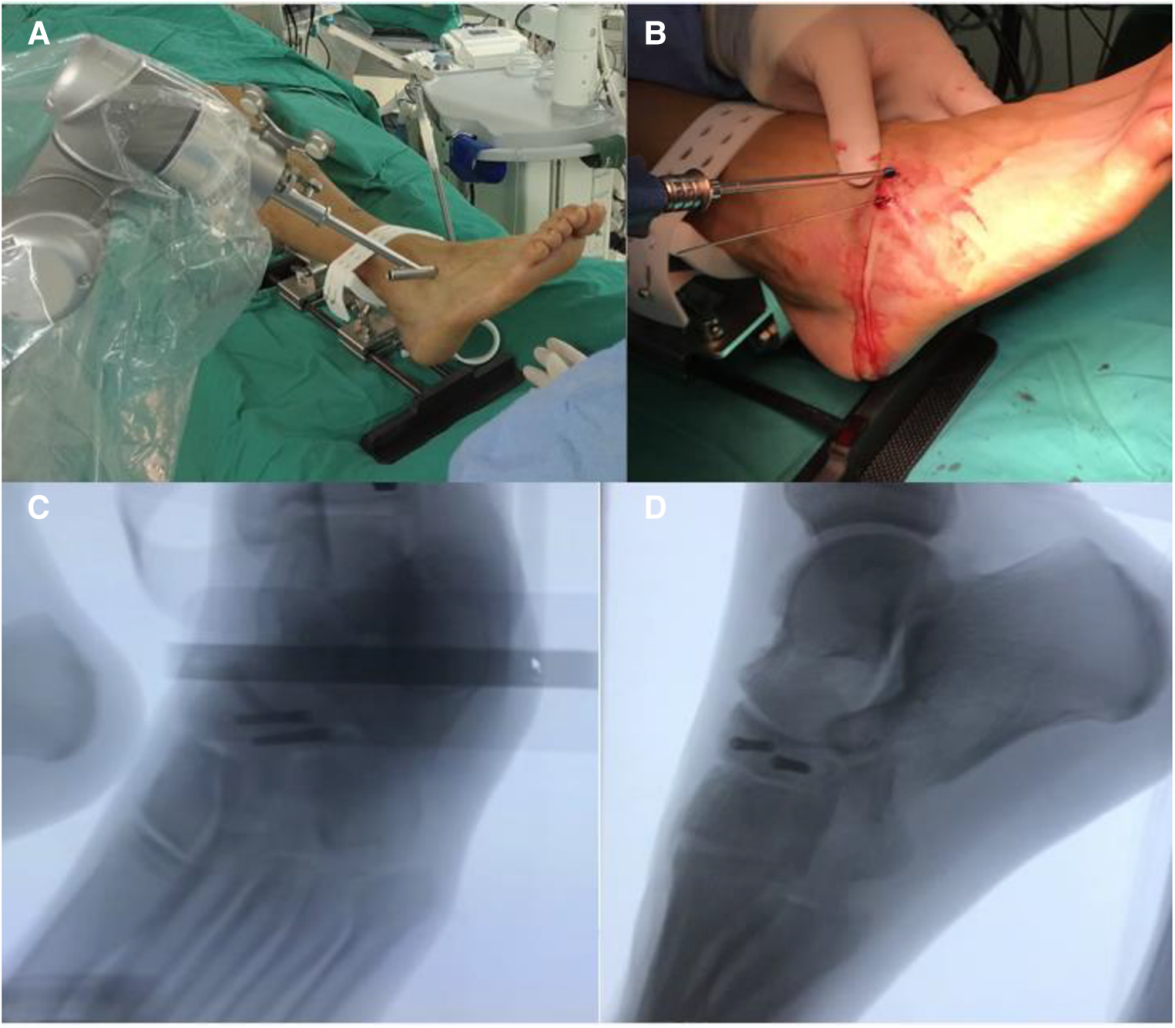
Intraoperative operation assisted by TINAVI intelligent orthopedic robot. (**A**) Intraoperative navigation assisted positioning by robot; (**B**) internal screw fixation *via* Kirschner wire prepositioning; (**C**) front view of the right foot of Artis zeego; (**D**) lateral view of the right foot of Artis zeego.

Patients were anesthetized with continuous epidural or general anesthesia in the supine position, and an inflatable tourniquet was routinely applied. An appropriate amount of venous blood was drawn from the median elbow vein and injected into a platelet-rich plasma (PRP) preparation device for centrifugation. After the onset of anesthesia, the ipsilateral hip is padded and the lower extremity is routinely disinfected and toweled. Activate the full TiRobot orthopaedic robot system and adjust the 3D motion measurement system. A 3D positioning and guidance frame is installed and the patient is properly immobilized to achieve stability of the affected foot and ankle joint. After proper fixation, a patient tracer is placed at the navicular bone and the affected foot is scanned fluoroscopically using the immediate intraoperative 3D imaging system to obtain 3D CT images. Then the TiRobot orthopaedic robot was used for intraoperative navigation, and the 3D CT was uploaded to the navigation workstation for processing. After reconstructing the cross-sectional, coronal, and sagittal images, the surgical site was positioned and a virtual surgical design was performed, with the virtual guide pin placed perpendicular to the fracture line in each plane at the mid-axis position of the navicular bone of the foot, thus planning the screw placement trajectory (Figure 3). After planning the path, navigation indicators were placed in the different orifices of the guiding frame, and the surgical entry point and the orientation of the guide pin were positioned by a robot-assisted fine tuning system with the assistance of an immediate intraoperative 3D imaging system. After positioning and locking, the navigation indicator is replaced with a sleeve and a 1.3 mm guide needle. The sleeve was gradually moved closer and two small incisions of 0.5–1 cm were made on the dorsum of the foot before the skin was applied and retracted to avoid thermal damage to the soft tissues. After patching, 2 guide needles were drilled into each of them, and the guide needles were correctly positioned using Artis zeego. The medulla was expanded *via* the guide pins and two 4.0 mm hollow nails (Acumed) were screwed into each of them to fix the navicular fracture. The fracture was repositioned and the position and length of the screws were satisfactory, and the guide pins were withdrawn using the immediate intraoperative 3D imaging system (Figure 4). The wound was rinsed and PRP was driven into the fracture at the incision.

• The surgical approach to the treatment of tarsal navicular fractures using ORIF is as follows.

The patient is placed in the supine position, and an appropriate amount of venous blood is drawn from the median elbow vein and injected into a platelet-rich plasma (PRP) preparation device for centrifugation. After the onset of anesthesia, the ipsilateral hip is padded and the lower extremity is routinely disinfected and toweled. For displaced dorsal avulsion fractures of the navicular bone >2 mm, a dorsal medial approach incision between the anterior and posterior tibial tendons is often used; for navicular tuberosity fractures, a medial approach is used; for navicular body fractures, a longitudinal dorsal incision between the lesser extensor tendon and the anterior tibial muscle is most commonly used because of the need to expose the talocrural and navicular cuneiform joints. The soft tissues of the superficial peroneal nerve, saphenous nerve, dorsalis pedis artery, great saphenous vein, anterior and posterior tibial tendons, and lesser extensor tendon are freed and retracted for protection. The periosteum and joint capsule are dissected and the fracture is completely exposed. The fracture site is cleared by chiseling out the dorsal tuberosity using a bone knife and removed with an occlusal forceps. For patients with osteosclerosis present, microfracture management is performed using a kerf pin. The fracture site was properly repositioned with a point repositioning forceps, then a sleeve was installed and two 1.3 mm guide pins were placed from the outside to the inside, respectively. C-arm machine fluoroscopy showed satisfactory guide pin position. Two 4.0 mm hollow screws were screwed through the guide pin reaming to fix the navicular fracture. Attention should be paid to the length of the screws to avoid unnecessary injury to the anterior and posterior tibial tendons. The fluoroscopy was repeated and the fracture was satisfactorily repositioned and the internal fixation device was withdrawn from the guide pin. The wound was irrigated and the surrounding soft tissue damage such as tendons, blood vessels and nerves was explored. PRP was inserted into the fracture break within the incision.

### Postoperative treatment

After surgery, the affected limb is elevated, the wound is treated with a dressing change within 2 weeks, and muscle contraction training and functional exercises for the toes, ankle, knee, and hip joints are performed. A short walking boot was used to immobilize the affected foot after surgery to provide stability and protection, as well as to facilitate protective observation of the skin condition. The incision sutures are removed at 2 weeks postoperatively and full range of motion training in bed is initiated. It is recommended that the walking boot be removed at rest for plantarflexion and dorsiflexion exercises to restore joint motion and prevent joint stiffness. Patients were instructed to limit weight bearing for 6 weeks and were allowed to gradually move down to partial weight bearing with the assistance of the walking boot and to strengthen midfoot joint mobility training according to the review. full weight bearing was allowed at 12 weeks and sports were allowed at 24–48 months. Patients were informed to visit the outpatient clinic for follow-up at 6, 12, 24 and 48 weeks postoperatively.

### Study visit

**Figure 5:**
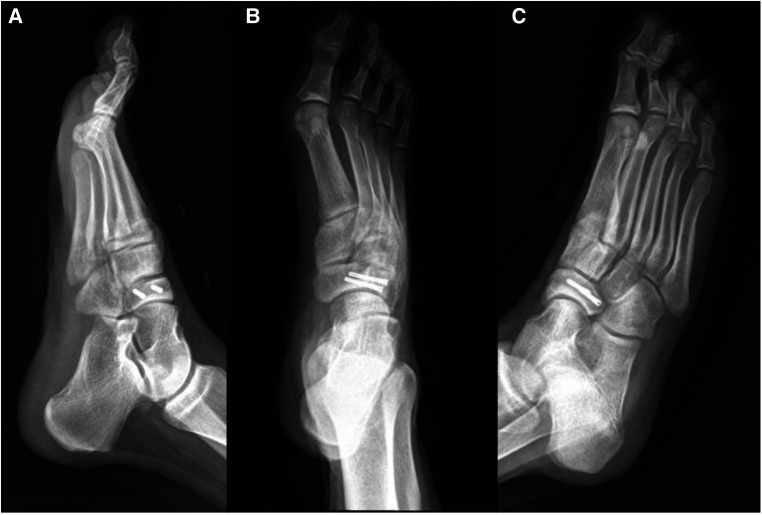
Immediate postoperative imaging data. (**A**) Non-loaded lateral x-ray of the right foot; (**B**) anteroposterior x-ray of the right foot; (**C**) oblique x-ray of the right foot.

**Figure 6:**
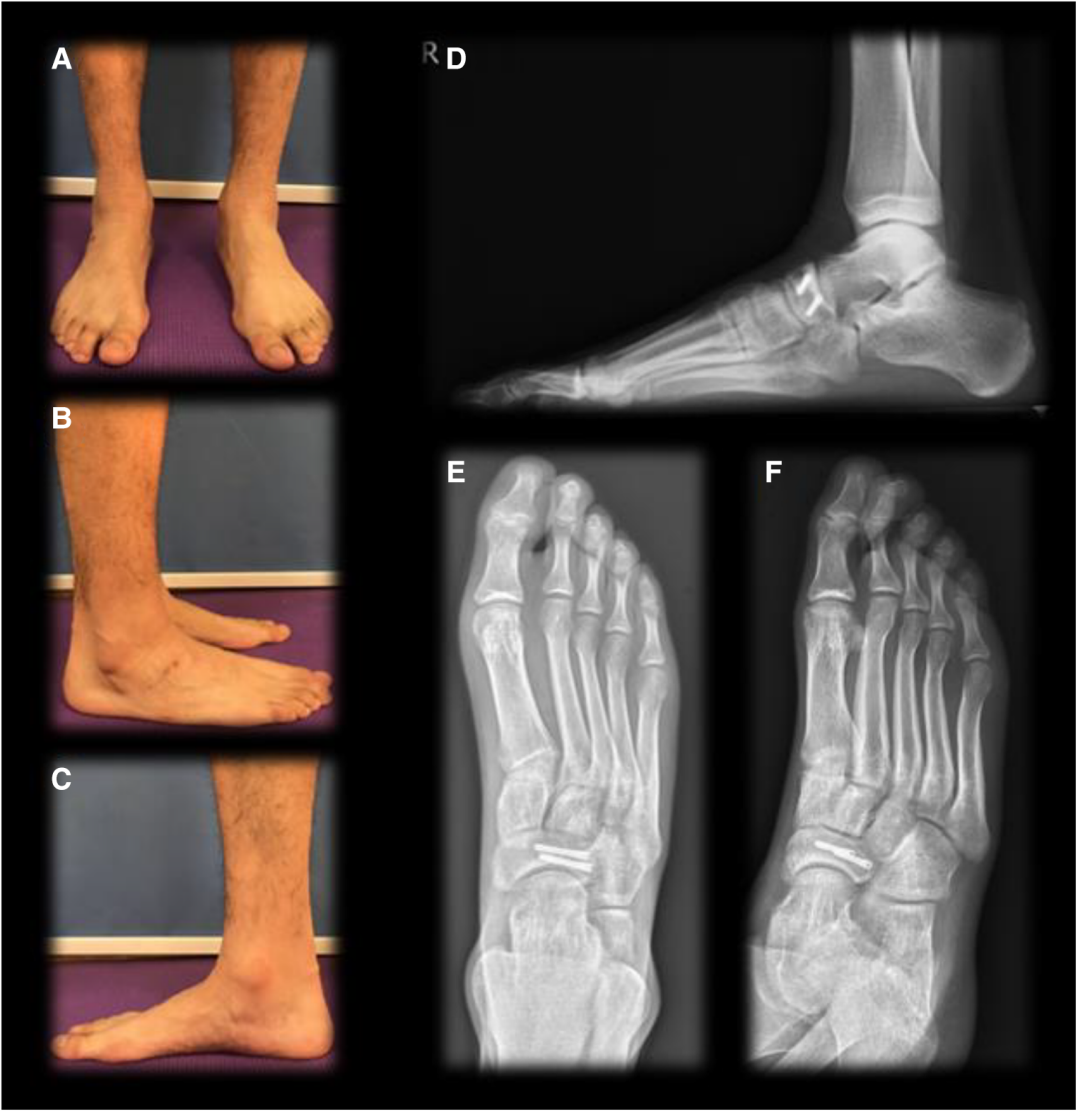
Twelve weeks postoperative follow-up. (**A**) Front view standing on two feet; (**B**) lateral view of the affected foot standing on two feet; (**C**) medial view of the affected foot standing on one foot; (**D**) weight-bearing lateral x-ray of the right foot; (**E**) anteroposternal x-ray of the right foot; (**F**) oblique x-ray of the right foot.

**Figure 7:**
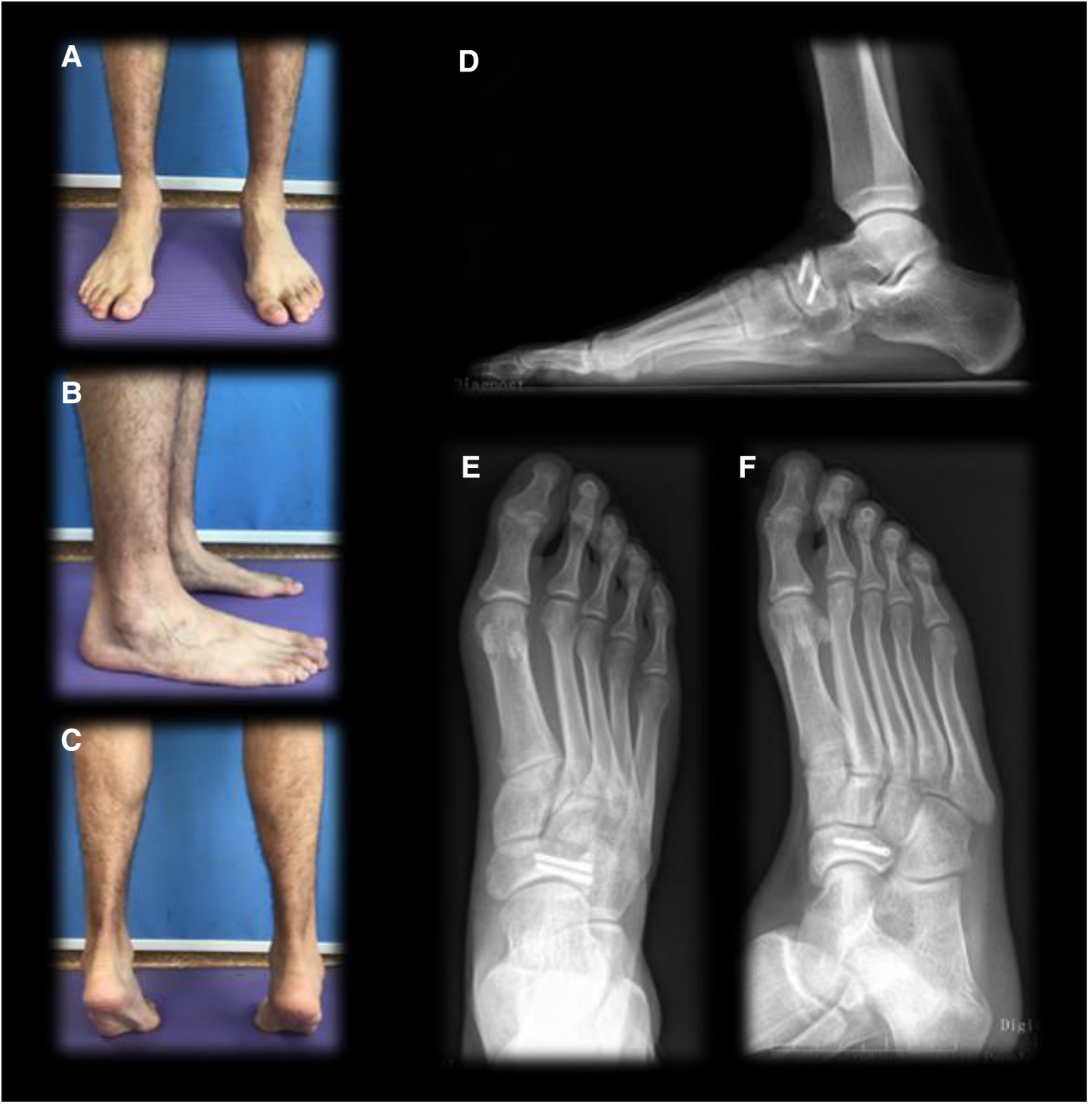
One year postoperative follow-up. (**A**) Front view standing on two feet; (**B**) lateral view of the affected foot standing on two feet; (**C**) rear view with both feet on tiptoe; (**D**) weight-bearing lateral x-ray of the right foot; (**E**) anteroposternal x-ray of the right foot; (**F**) oblique x-ray of the right foot.

Each patient was followed up for at least 1 year after surgery and came to the outpatient clinic for review at the prescribed time. In addition to undergoing a standardized physical examination of the affected limb, patients were required to undergo a series of imaging examinations to assess fracture healing (Figures 5–7). Finally, patients were instructed to complete the AOFAS ankle/hindfoot score as well as a questionnaire for the VAS score.

### Statistical analysis

The statistical analyses were performed with SPSS version 26.0. Quantitative data conforming to normal distribution were expressed as mean ± standard deviation (X¯ ± s). Paired-sample *t*-test was used to compare quantitative data within the same group before and after surgery, and independent-sample *t*-test was used to compare quantitative data between two groups. The difference was considered statistically significant at *p* < 0.05.

## Results

### Clinical and radiographic outcomes

Follow-up was obtained in all 27 patients after surgery, with a mean follow-up time of 21.81 months, ranging from 15 to 29 months (Table 1). Regarding the baseline data, the length of stay, time on the floor and fracture healing time were slightly shorter in the intraoperative robot-assisted group than in the conventional ORIF group, but there was no statistical difference between the two groups (*p* > 0.05) (Table 2). Significant improvements in clinical function were observed in both groups at the final follow-up (Tables 3, 4). The final VAS in the intraoperative robot-assisted group (0.75 ± 0.25) was significantly lower than the preoperative VAS (7.00 ± 0.41) (*p* < 0.001), and the final AOFAS (92.25 ± 2.22) was significantly higher than the preoperative AOFAS (46.75 ± 2.63) (*p* < 0.001); the final VAS in the ORIF group (0.50 ± 0.29) was significantly lower than preoperative VAS (7.00 ± 1.41) (*p* < 0.001), and last AOFAS (82.25 ± 7.15) was significantly higher than preoperative AOFAS (44.50 ± 7.05) (*p* < 0.001). Also, the functional scores of AOFAS in the intraoperative robot-assisted group were statistically superior to those in the ORIF group at the last follow-up (*p* = 0.008), while there was no statistical difference in VAS pain scores between the two groups (*p* > 0.05).

**Table 1:** Demographic data.

	Total (*n* = 27)	Range	RA (*n* = 7)	Range	ORIF (*n* = 20)	Range
Age	35.07 ± 10.37	14–48	31.43 ± 7.53	16–37	36.35 ± 11.08	14–48
Gender						
Males	12		3		9	
Females	15		4		11	
Violence						
Sprain	10		4		6	
Fall	13		3		10	
Traffic accident	4		0		4	
Side						
Left	12		3		9	
Right	14		4		10	
Both	1		0		1	
Sangeorzan type						
1	0		0		0	
2	4		0		4	
3	23		7		16	
Internal fixation						
Remaining	21		6		15	
Removal	6		1		5	
Time to WB (weeks)	9.30 ± 2.93	4–14	8.57 ± 2.99	4–12	9.55 ± 2.95	5–14
Time to healing (weeks)	17.78 ± 5.53	8–28	15.14 ± 5.40	8–24	18.70 ± 5.40	10–28

RA, robot-assisted; ORIF, open reduction internal fixation; WB, weight bearing.

**Table 2:** Comparison of follow-up data between two groups (`x ± s).

	Hospitalization (days)	Time to WB (weeks)	Time to healing (weeks)
RA (*n* = 7)	4.86 ± 0.90	8.57 ± 2.99	15.14 ± 5.40
ORIF (*n* = 20)	5.15 ± 0.22	9.55 ± 2.95	18.70 ± 5.40
*t* value	−0.689	−1.500	−0.753
*p* value	0.497	0.146	0.458

**Table 3:** Comparison of AOFAS score before and after operation (`x ± s).

	RA (*n* = 7)	ORIF (*n* = 20)	*t* value	*p* value
Preoperative	46.75 ± 2.63	44.50 ± 7.05	−0.780	0.443
The last follow-up	92.25 ± 2.22	82.25 ± 7.15	2.909	0.008
*t* value	−20.72	−24.72		
*p* value	<0.01	<0.01		

**Table 4:** Comparison of VAS score before and after operation (`x ± s).

	RA (*n* = 7)	ORIF (*n* = 20)	*t* value	*p* value
Preoperative	7.00 ± 0.41	7.00 ± 1.41	−0.171	0.866
The last follow-up	0.75 ± 0.25	0.50 ± 0.29	−0.279	0.782
*t* value	13.49	28.79		
*p* value	<0.01	<0.01		

### Complications

In terms of complications, a total of 27 patients in the intraoperative robot-assisted group and the ORIF group were operated successfully, and no intraoperative complications occurred. In the ORIF group, two cases of dermal nerve injury occurred, manifested as numbness, pain, and abnormal sensation, which were considered to be intraoperative dermal nerve injury and gradually relieved by oral nutritional drugs. Other short-term complications, such as wound infection and failure of internal fixation, did not occur. In addition to these, no long-term complications such as fracture non-union, delayed healing, ischemic osteonecrosis and traumatic arthritis were observed in any of the postoperative imaging data. A total of 6 patients had their implants removed later on a voluntary basis, including 1 in the intraoperative robot-assisted group and 5 in the ORIF group.

## Discussion

The treatment of a navicular fracture depends not only on the type and extent of the fracture, but also on the functional requirements of the patient (10). Displaced navicular body fractures and nodal avulsion fractures often require surgical treatment, including joint mismatch >1 mm, medial column shortening >2–3 mm, open fractures, and nondisplaced navicular body fractures that have failed to respond to conservative treatment for >6 weeks (9, 14). Surgical treatment of navicular fractures is not only to achieve repositioning and fixation of the navicular fracture end, but also to restore the length and stability of the medial column and to restore the integrity of the articular surface (3). ORIF is the gold standard for displaced navicular fractures and talocalcaneal joint destruction (15). ORIF is the gold standard for navicular fracture displacement and talocalcaneal joint destruction. The commonly used approaches are the dorsal central incision, double incision and anteromedial incision, which may have complications related to skin healing and fracture healing (3, 9). The current difficulties of ORIF operation are inaccurate intraoperative positioning, trauma, slow postoperative recovery, and increased patient pain (13). On the other hand, the complex three-dimensional structure of the navicular bone on x-rays overlaps with the surrounding bones, which greatly increases the difficulty of diagnosing navicular fractures (16). Due to the special anatomical structure and location of the navicular bone, closed reduction percutaneous screw fixation is still challenging and not yet popular in clinical practice (3, 4, 10). In our study, for displaced navicular fractures, we tried to perform early closed reduction and internal fixation with the assistance of robot. Intraoperative robot has been reported and rapidly developed in some orthopaedic fields, which can improve surgical precision and shorten the length of x-ray exposure for the surgeon. However, most of the robot systems at home and abroad are used in trauma, spine, and joint fields for some larger skeletal joints, and their application in smaller skeletal joints is still in the basic research stage (17, 18).

The small size and complex structure of the tarsal navicular bone requires high surgical precision, and therefore has special requirements for the surgical procedure as well as design and application of robot systems. We recommend that each patient be given a CT scan 3D reconstruction preoperatively to adequately refine preoperative planning in order to reduce intraoperative planning time, operative time and tourniquet time. CT scan 3D reconstruction is also important for the design of the surgical plan, the choice of incision and fixation. The TINAVI intelligent orthopaedic robot (TINAVI Medical Technologies, Beijing, China) is an intelligent sub-millimeter robot capable of being applied to limb, acetabular, pelvic fracture and full segment spine surgery, with a powerful optical tracking system and a stable robotic arm for surgical navigation and positioning. In this study, we not only collected complete preoperative imaging data of patients, but also used the TINAVI intelligent orthopaedic robot for intraoperative navigation and positioning, which made the fracture part location nailing more accurate, shortened the operation time while assisting minimally invasive surgical treatment, reduced surgical trauma, improved stability, and facilitated the rapid recovery of patients. Our study also used the Artis zeego immediate intraoperative 3D imaging system, which can obtain CT scanning intraoperatively, equipped with the most advanced clinical functions, combining the accuracy of intraoperative fluoroscopy and the integrity of dynamic 3D scanning, showing the fracture line morphology of the navicular bone more intuitively and stereoscopically, and providing a reference for real-time intraoperative navigation. It provides an accurate and efficient integrated platform for clinicians’ preoperative diagnosis, intraoperative planning, and postoperative evaluation. With the aid of intraoperative robot, the guide pins are placed in a single pass, which reduces the side injuries caused by repeated intraoperative adjustment of the guide pins; the surgical planning is more detailed and comprehensive, which allows for more confident minimally invasive surgery; the number of fluoroscopic views is significantly reduced, and the overall repositioning of the fracture line and joint surface can be observed. It should be noted that although intraoperative robot allows for perfect localization of the fracture site, operator manipulation and skeletal micromotion will inevitably affect this process to a greater or lesser extent. Therefore, when performing intraoperative robot and percutaneous screw fixation of the navicular bone, the use of a three-dimensional positioning and guidance framework to maintain stability of the foot and ankle is essential for accurate nail placement.

In this study, 7 patients underwent intraoperative robot-assisted percutaneous screw internal fixation and 20 patients underwent ORIF, and the results were satisfactory in both groups. After comparison, we found that the intraoperative robot-assisted group showed some reduction in hospital days, time to weight bearing and fracture healing time compared to the ORIF group, although the statistical difference was not significant. This may imply a potential role of the use of intraoperative robot for preoperative planning and postoperative recovery. At the final follow-up, the AOFAS scores and VAS scores of patients in both groups showed a significant improvement compared to the preoperative period, with some statistical and clinical significance. Among them, the AOFAS scores in the intraoperative robot-assisted group were higher than those in the ORIF group at the final follow-up, while there was no statistical difference in the VAS scores, confirming the relief of painful symptoms of tarsal navicular fracture by surgical treatment and tentatively indicating that the intraoperative robot-assisted group could better help the improvement of joint function, further suggesting that the improvement of surgical precision by intraoperative robot could contribute to the improvement of functional activities such as postoperative joint movement and support. This may have some correlation with earlier weight bearing on the floor. At follow-up, we noted that patients in the intraoperative robot-assisted group were more satisfied with the incision, which allowed them to attempt earlier joint motion and functional exercise. In conclusion, intraoperative robot-assisted closed reduction percutaneous screw internal fixation of the navicular fracture of the foot allows for precise positioning, one nail placement, both less invasive and aesthetically pleasing, relief of pain symptoms, as well as better joint function.

Although tarsal navicular fractures have a high healing rate, they are prone to a range of complications that can affect functional prognosis (8, 14, 19). The common complications include traumatic arthritis, joint stiffness, postoperative residual pain, and ischemic osteonecrosis, among which traumatic arthritis is the most common complication after navicular fracture (3, 9, 20). To eliminate heterogeneity, our study excluded patients with comminuted navicular body fractures who had to be considered for talocalcaneal fusion. In the study by Coulibaly et al., a total of 35 cases of traumatic arthritis, 3 cases of infection, 2 cases of osteochondral nonunion, 1 case of osteofascial compartment syndrome, 1 case of deep vein thrombosis and 1 case of complex regional pain syndrome (CRPS) were seen in 41 navicular fractures that underwent ORIF (13). In this study, two patients in the ORIF group developed numbness, pain, sensory abnormalities and other symptoms of dermal nerve injury at follow-up, but no other complications were reported, which suggests that we need to pay more attention to the protection of soft tissues during the surgical incision and operation in the future. A total of six patients underwent secondary surgery to remove the internal fixation after surgery, including one case in the intraoperative robot-assisted group and five cases in the ORIF group.

### Limitations of the study

Limitations of this study include retrospective nature and small sample size. Firstly, there was some recall bias in this study due to a retrospective case series report. Secondly, solitary tarsal navicular fractures are relatively rare, and the small sample size resulted in unreliable results, which can only serve as a preliminary attempt and exploration of the research team in the field of intraoperative robot. Thirdly, in this study only relatively simple fractures were selected for robot-assisted closed reduction percutaneous screw internal fixation, and comminuted navicular fractures with difficult repositioning could not be the target of this technique. A larger multicenter study, as well as a prospective randomized trial, is needed to improve the surgical procedure in order to make a more objective and comprehensive evaluation of this procedure and guide the subsequent treatment of tarsal navicular fractures.

## Conclusion

In conclusion, this small series of studies obtained relatively good clinical results. The advantage of Intraoperative robot-assisted closed reduction and percutaneous internal fixation for tarsal navicular fracture is that it avoids the orthopaedic surgeon's uncertainty about the quality of the repositioning and implant placement, as well as reducing soft tissue damage, operative time, learning curve and potential complications. This study initially validated the feasibility of intraoperative robot-assisted closed reduction internal fixation of tarsal navicular fractures in the treatment of tarsal navicular fractures, with a view to providing ideas for subsequent minimally invasive and precise treatment in traumatic orthopaedics.

## Data Availability

The original contributions presented in the study are included in the article/Supplementary Material, further inquiries can be directed to the corresponding author/s.
